# Human Neural Stem Cells Encoding ChAT Gene Restore Cognitive Function via Acetylcholine Synthesis, Aβ Elimination, and Neuroregeneration in APPswe/PS1dE9 Mice

**DOI:** 10.3390/ijms21113958

**Published:** 2020-05-31

**Authors:** Dongsun Park, Ehn-Kyoung Choi, Tai-Hyoung Cho, Seong Soo Joo, Yun-Bae Kim

**Affiliations:** 1 Department of Biology Education, Korea National University of Education, Cheongju, Chungbuk 29173, Korea; dvmdpark@knue.ac.kr; 2 Central Research Institute, Designed Cells Co., Ltd., Cheongju, Chungbuk 28576, Korea; ekchoi@designedcells.com; 3 Department of Neurosurgery, Korea University College of Medicine, Korea University Anam Hospital, Seoul 02841, Korea; choth2@korea.ac.kr; 4 Department of Marine Molecular Biotechnology, Gangneung-Wonju National University, Gangneung, Gangwon 25457, Korea; ssj66@gwnu.ac.kr; 5 College of Veterinary Medicine and Veterinary Medical Center, Chungbuk National University, Cheongju, Chungbuk 28644, Korea

**Keywords:** Alzheimer disease, human neural stem cell, choline acetyltransferase, acetylcholine, Aβ deposition, neuroregeneration

## Abstract

In Alzheimer disease (AD) patients, degeneration of the cholinergic system utilizing acetylcholine for memory acquisition is observed. Since AD therapy using acetylcholinesterase (AChE) inhibitors are only palliative for memory deficits without slowing or reversing disease progress, there is a need for effective therapies, and stem cell-based therapeutic approaches targeting AD should fulfill this requirement. We established a human neural stem cell (NSC) line encoding choline acetyltransferase (ChAT) gene, an acetylcholine-synthesizing enzyme. APPswe/PS1dE9 AD model mice transplanted with the F3.ChAT NSCs exhibited improved cognitive function and physical activity. Transplanted F3.ChAT NSCs in the AD mice differentiated into neurons and astrocytes, produced ChAT protein, increased the ACh level, and improved the learning and memory function. F3.ChAT cell transplantation reduced Aβ deposits by recovering microglial function; i.e., the down-regulation of β-secretase and inflammatory cytokines and up-regulation of Aβ-degrading enzyme neprilysin. F3.ChAT cells restored growth factors (GFs) and neurotrophic factors (NFs), and they induced the proliferation of NSCs in the host brain. These findings indicate that NSCs overexpressing ChAT can ameliorate complex cognitive and physical deficits of AD animals by releasing ACh, reducing Aβ deposit, and promoting neuroregeneration by the production of GFs/NFs. It is suggested that NSCs overexpressing ChAT could be a candidate for cell therapy in advanced AD therapy.

## 1. Introduction

Alzheimer disease (AD) is a progressive neurodegenerative disease, which is characterized by degeneration and the loss of neurons and synapses throughout the brain. Memory and cognitive functions of AD patients progressively decline, and patients become demented and die prematurely [1]. In AD patients, dysfunction of the cholinergic system is one of the causes of cognitive and non-cognitive disorders where decreased activity of choline acetyltransferase (ChAT), an enzyme responsible for acetylcholine (ACh) synthesis, is noted [2]. For AD therapy, the administration of acetylcholinesterase (AChE) inhibitors partially recovers cognitive deficits [3]. Since these drugs are only palliative without slowing or reversing disease progress, there is a need for effective therapies for patients with AD, and stem cell-based therapeutic approaches targeting AD should fulfill this requirement.

As an animal model of human AD, APPswe/PS1dE9 transgenic (Tg) mice are characterized by amyloid β (Aβ) plaques, neurofibillary tangles, and extensive inflammation leading to ACh depletion and learning and memory impairments [4]. In APPswe/PS1dE9 mice, functional changes of microglia; i.e, the up-regulation of Aβ-producing systems (cytokines and β-secretase) and down-regulation of Aβ-clearing machinery (scavenger receptors and degrading enzymes such as neprilysin) play a key role for accumulation of neurotoxic Aβ peptides [5].

Recently, we established F3.ChAT, a human neural stem cell (NSC) line encoding human ChAT gene, and demonstrated its effectiveness in the improvement of cognitive function in animals with kainic acid-induced hippocampal injury [6], AF64A-induced cholinergic neuronal degeneration [7], and *N*-methyl-D-aspartate (NMDA)-induced amygdala injury [8] by increasing brain ACh levels. In addition, F3.ChAT NSCs improved the cognitive function and physical activity of aging mice and middle cerebral artery occlusion (MCAO)-challenged rats, and they notably increased the brain level of microtubule-associated protein 2 (MAP2), a cytoskeletal protein of neurons [9,10], indicative of a restoring activity of the stem cells on the neuronal integrity. Furthermore, it is well known that stem cells exert anti-inflammatory and immunomodulatory activities in many central nervous system (CNS) diseases including neonatal hypoxic-ischemic encephalopathy [11] and multiple sclerosis [12]. Such results led us to investigate the effectiveness of F3.ChAT NSCs on the cognitive and physical functions of APPswe/PS1dE9 mice and underlying mechanisms, especially focusing on the Aβ elimination and neuroregeneration.

## 2. Results

### 2.1. Cholinergic Properties of NSCs

F3.ChAT NSCs displayed higher expression of cholinergic system markers than their parental F3 NSCs, including ChAT, high-affinity choline transporter 1 (ChT1), vesicular ACh transporter (VAChT), and nicotinic ACh receptors (AChRs), while AChE, an ACh-degrading enzyme, was down-regulated (Supplementary Figure S1).

### 2.2. Distribution and Differentiation of NSCs

**Figure 1:**
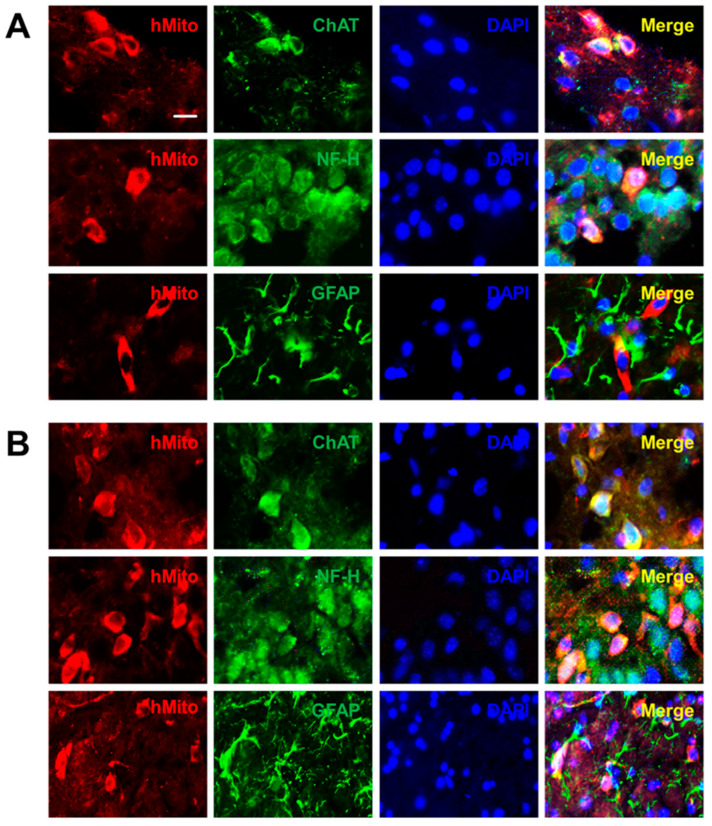
Representative immunohistochemical findings on the differentiation and production of functional protein (ChAT) of transplanted neural stem cells (NSCs). Differentiation of human mitochondria (hMito)-positive F3 ((**A**): Group 3) and F3 NSC line overexpressing ChAT gene (F3.ChAT) ((**B**): Group 4) into neurons (NF-H-positive) and astrocytes (GFAP-positive), and ChAT production were analyzed by double immunostaining in the hippocampus of APPswe/PS1dE9 transgenic mice. GFAP: glial fibrillary acidic proteins. Bar = 10 μm.

Four weeks after intracerebroventricular (ICV) transplantation of NSCs (10^6^ cells/mouse) into the brain of APPswe/PS1dE9 Tg mice, transplanted F3 and F3.ChAT human (hMito-positive) NSCs were detected in the hippocampus and cortex. In double immunostaining, both F3 (Figure 1A) and F3.ChAT (Figure 1B) NSCs were found to differentiate into neurons (NF-H-positive) and in part into astrocytes (GFAP-positive) in the brain microenvironment, and they produced ChAT protein. By comparison, the number of F3.ChAT cells (89.2 cells/mm^2^) observed in the brain was more than F3 cells (59.4 cells/mm^2^) (Supplementary Figure S2A), and notably, a higher ratio of transplanted F3.ChAT cells than F3 cells differentiated into neurons and produced the ChAT protein (Supplementary Figure S2B).

### 2.3. Recovery of Cholinergic and Cognitive Functions

**Figure 2:**
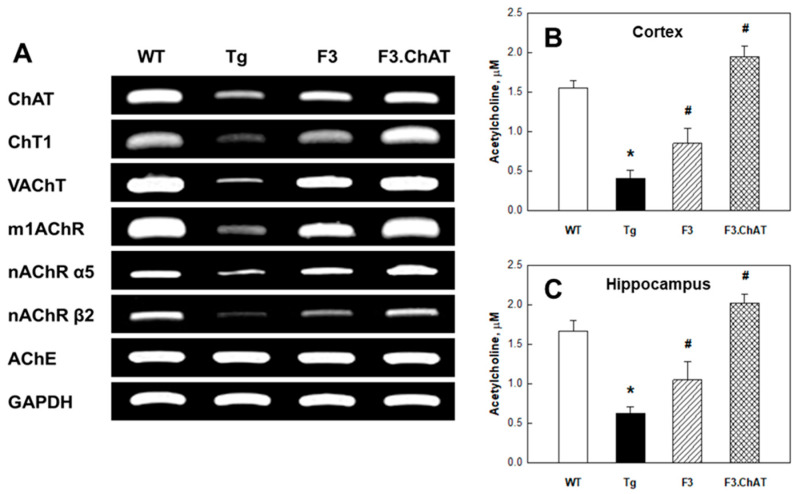
Cholinergic nervous system markers and acetylcholine concentration in the mouse brain. (**A**): Cholinergic nervous system markers. (**B**,**C**): Acetylcholine concentration in the cortex (**B**) and hippocampus (**C**) of wild-type (WT) and APPswe/PS1dE9 transgenic (Tg) mice transplanted with F3 or F3.ChAT neural stem cells. * Significantly different from WT mice (*p* < 0.05). ^#^ Significantly different from Tg mice (*p* < 0.05).

**Figure 3:**
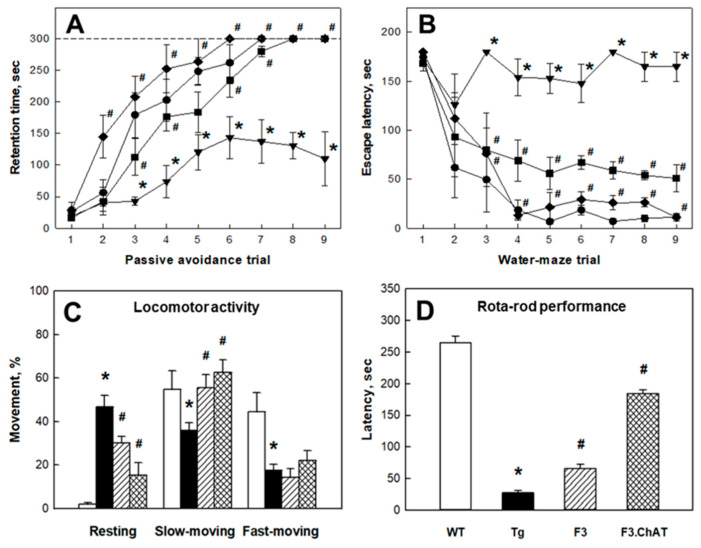
Cognitive function and physical activity of wild-type (WT) and APPswe/PS1dE9 transgenic (Tg) mice transplanted with F3 or F3.ChAT neural stem cells (NSCs). (**A**,**B**): Passive avoidance performance (A) and Morris water-maze performance (B). The endpoint was set to 300 s, if the animals failed to find the platform. ●: WT, ▼: Tg, ■: Tg + F3 NSCs, ♦: Tg + F3.ChAT NSCs. (**C**,**D**): Locomotor activity (C) and rota-rod performance (D). White: WT, black: Tg, shaded: Tg + F3 NSCs, checked: Tg + F3.ChAT NSCs. * Significantly different from WT mice (*p* < 0.05). ^#^ Significantly different from Tg mice (*p* < 0.05).

In the APPswe/PS1dE9 Tg mouse brain, the expression of cholinergic functional genes such as ChAT, ChT1, VAChT, and AChRs (m1AChR, nAChR α5, and nAChR β2) associated with the synthesis, transportation, and reception of ACh markedly decreased (Figure 2A). Hence, such degenerative changes in the cholinergic system might led to the decreased ACh levels in both the cortex (Figure 2B) and hippocampus (Figure 2C), resulting in deficits of cognitive function (Figure 3A,B) and neurobehavioral activities (Figure 3C,D). Interestingly, ICV transplantation of F3 or F3.ChAT cells significantly improved the learning and memory functions in passive avoidance and water-maze performances as well as neurobehavioral activities in locomotor and rota-rod performances 4 weeks post-transplantation. Such recoveries of cognitive and behavioral functions were in parallel with the increased levels of brain ACh following NSC transplantation (Figure 2B,C), in which F3.ChAT cells were superior to F3 parental cells. In fact, transplanted NSCs produced ChAT protein (Figure 1A,B) and notably increased host cholinergic system markers (Figure 2A).

### 2.4. Aβ Elimination via Microglial Function Restoration

**Figure 4:**
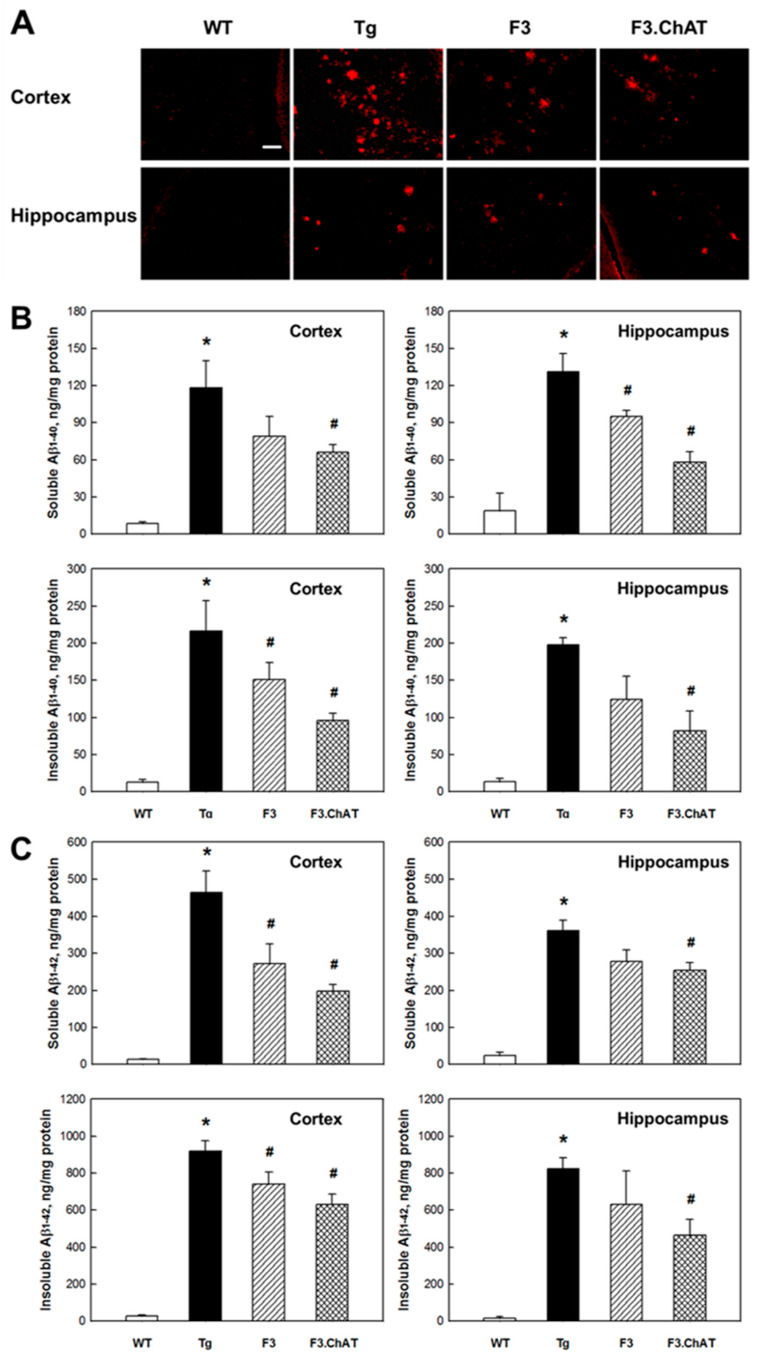
Amyloid β deposit and plaque formation in the brain of wild-type (WT) and APPswe/PS1dE9 transgenic (Tg) mice transplanted with F3 or F3.ChAT neural stem cells. (**A**): Aβ (senile) plaques in the cortex and hippocampus. (**B**,**C**): Concentration of soluble and insoluble Aβ_1-40_ (**B**) and Aβ_1-42_ (**C**) peptides in the cortex and hippocampus of WT and Tg mice. Bar = 20 μm. * Significantly different from WT mice (*p* < 0.05). ^#^ Significantly different from Tg mice (*p* < 0.05).

In APPswe/PS1dE9 mice, amyloid plaques were found in the cortex and hippocampus (Figure 4A). The amounts of SDS-soluble and SDS-insoluble Aβ_1-40_ and Aβ_1-42_ peptides were much higher than those in wild-type animals (Figure 4B,C). Notably, such Aβ deposition and plaque formation were markedly reduced by transplantation of NSCs, in which F3.ChAT cells were more effective than F3 cells.

As one of the key factors for Aβ formation, microglial dysfunctions were confirmed in 19-month- old APPswe/PS1dE9 mice; i.e., levels for BACE, tumor-necrosis factor-α (TNF-α), and interleukin-1β (IL-1β) increased, while levels for neprilysin, CD11b, scavenger receptor A (SRA), SRB1, and receptor for advanced glycation end products (RAGE) decreased (Supplementary Figure S3A). However, interestingly, the altered microglial functions in the AD mice restored to the levels of wild-type animals following the transplantation of F3.ChAT NSCs, notably wherein neprilysin, an Aβ-degrading enzyme, was over-produced. As an example of reactivated microglia, CD11b-positive microglial cells were observed around the amyloid plaques in the brain of F3.ChAT cell-grafted APPswe/PS1dE9 Tg mice (Supplementary Figure S3B).

### 2.5. Neuroregeneration Mediated by GFs/NFs

**Figure 5:**
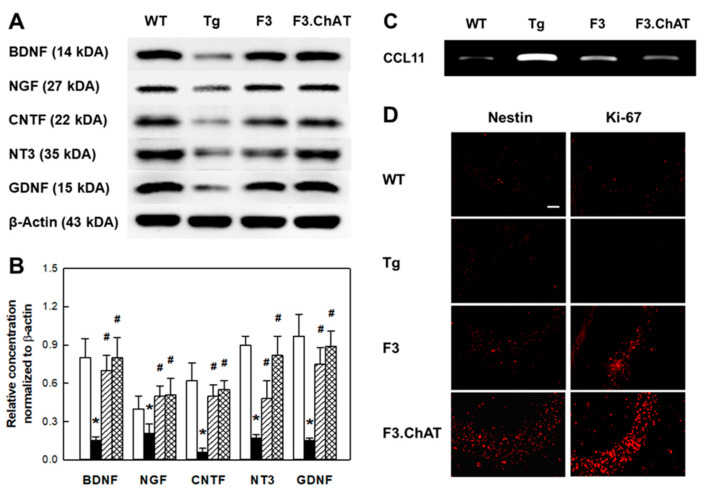
Production of growth and neurotrophic factors, expression of CCL11, and host stem cell proliferation in the brain of wild-type (WT) and APPswe/PS1dE9 transgenic (Tg) mice transplanted with F3 or F3.ChAT neural stem cells. (**A**,**B**): Growth and neurotrophic factor production. White bars: WT mice, black bars: Tg mice, shaded bars: Tg mice transplanted with F3 cells, checked bars: Tg mice transplanted with F3.ChAT cells. (**C**): CCL11 (a chemokine known as eotaxin) expression. (**D**): Representative findings of host neural stem cells (nestin-positive) and proliferating cells (Ki-67-positive) in the hippocampus of WT and Tg mice. Bar = 20 μm. * Significantly different from WT mice (*p* < 0.05). ^#^ Significantly different from Tg mice (*p* < 0.05).

In Western blot analysis of AD Tg mouse brain, markedly decreased levels of growth factors/neurotrophic factors (NFs/NFs) (Figure 5A,B) and proteins related to their signaling pathway (Supplementary Figure S4) were found compared to wild-type animals. However, the transplantation of NSCs restored the levels of CNTF and GDNF that were known to suppress cytokine expression, as shown in Supplementary Figure S3A. GFs/NFs of the neurotrophin family possessing neuroprotective activity and related to cholinergic innervation such as NGF, BDNF, and NT3 were also near-fully recovered by F3.ChAT transplantation. In addition, the signaling molecules for GFs/NFs including TrkB, PKC, CaMKII, p-AKT, and p-Erk were fully restored, too (Supplementary Figure S4).

In APPswe/PS1dE9 mice, the expression of CCL11 (eotaxin), a chemokine suppressing neurogenesis in aged animals, greatly increased (Figure 5C). While in NSC-grafted APPswe/PS1dE9 mice, CCL11 expression was markedly down-regulated, and the number of nestin-positive host NSCs exhibiting Ki-67 immunoreactivity was found to increase considerably (Figure 5D).

## 3. Discussion

In the present study, transplanted F3.ChAT NSCs in APPswe/PS1dE9 mouse brain were found to differentiate into neurons and astrocytes, produce the ChAT protein, increase the ACh level, and improve the learning and memory function. F3.ChAT cell transplantation reduced Aβ deposits by recovering microglial function such as the down-regulation of β-secretase and inflammatory cytokines and the up-regulation of Aβ-degrading enzyme neprilysin. F3.ChAT NSCs also restored GFs/NFs and induced the proliferation of NSCs in the host brain.

F3.ChAT NSCs encoding the human ChAT gene via a retroviral vector displayed an increased expression of genes related to ACh synthesis and secretion, while the ACh-degrading AChE gene was down-regulated ( Supplementary Figure S1). F3.ChAT NSCs could be an ideal model of stem cells for replacement therapy for a degenerative cholinergic system. In addition, F3.ChAT NSCs showed higher survivability and capacity to produce the ChAT protein for ACh synthesis in the brain microenvironment (Supplementary Figure S2A,B). Such higher survivability and neuronal differentiation of F3.ChAT cells than F3 parental cells were also confirmed in other AD animal models [6,7], which may be due to the increased production of neuroprotective and anti-inflammatory GFs/NFs including VEGF, GDNF, NGF, and CNTF in F3.ChAT cells [10].

Previous studies have reported that the expression of genes associated with cholinergic function such as ChAT, VAChT, and AChE markedly decreased in the brain of APPswe/PS1dE9 mice [13,14], and the expression of ChT1 and AChRs was also found to be reduced (Figure 2A) as shown in this study. Brain transplantation of F3.ChAT NSCs in the APPswe/PS1dE9 mice restored the genes of cholinergic system at the levels of wild-type mice. Muscarinic and nicotinic receptors are widely distributed in the hippocampus and cortex; they play key roles in learning and spatial/working memories [15,16], and ChAT is one of the well-known markers of motor neurons [17]. It appears that the improvements of cognitive and motor functions by transplantation of F3 or F3.ChAT cells might be due to the increased ACh levels in the brain (Figure 2B,C) originated not only from NSCs, but also from restored host cholinergic neurons.

Aβ peptides play a key pathogenic role in AD [18], and the deposition of Aβ is related with microglial dysfunction [19]. During the aging of APPswe/PS1dE9 mice, microglial cells undergo functional changes, leading to defective Aβ-clearance pathways and the abnormal accumulation of Aβ peptides [5,20]. Aβ peptides can activate microglia to produce cytokines and neurotoxins, hence promoting neurodegeneration [21]. In turn, inflammatory cytokines augment Aβ formation; TNF-α and IL-1β induce microglial dysfunction including the up-regulation of BACE, which is a key rate-limiting enzyme initiating Aβ formation [22,23]. In addition, the cytokines down-regulate Aβ-clearance/phagocytosis machinery such as Aβ-degrading enzymes neprilysin and matrix metalloproteinase 9 as well as Aβ receptors including SRA, CD36, and RAGE [5,24]. In the present study, we demonstrated that the transplanted NSCs recovered the original function of microglia; i.e., Aβ-clearing activity without excessive Aβ formation and aggravating inflammatory response (Supplementary Figure S3). Interestingly, the distribution and differentiation of NSCs in AD mouse brain were found to be triggered by chemoattractants such as TNF-α and several GFs/NFs released from the inflammatory tissues [5,6], as confirmed from the CD11b-positive microglial cells observed around the amyloid plaques (Supplementary Figure S3B).

Previous studies have reported immunomodulatory properties of NSCs in CNS diseases including AD [25] and experimental autoimmune encephalomyelitis [26]. It was found that neurotrophic factors including CNTF and GDNF from stem cells can regulate immune response [27,28]. CNTF stimulates the secretion of GDNF that reduces IL-1β and TNF-α expression [28,29]. Therefore, it is believed that the recovery of CNTF and GDNF by the transplantation of NSCs (Figure 5A,B) might down-regulate the inflammatory cytokine production (Supplementary Figure S3).

Neurotrophin family molecules including NGF and BDNF promote the survival of developing cholinergic neurons and reduces neuronal loss following excitotoxic insults [30,31]. NGF and BDNF are also involved in the cholinergic innervation and activity of ChAT in the hippocampus, and they were found to increase cognitive function in aged animals [32,33]. In our previous study, it was found that F3 NSCs express GFs/NFs including NGF, BDNF, NT3, and GDNF [34]. In addition, NSCs improved the cognitive function of AD model animals by enhancing hippocampal synaptic density mediated by BDNF [35,36], and F3 NSCs overexpressing NGF (F3.NGF) significantly restored the cognitive function of rats with ibotenic acid-induced hippocampal injury [37]. In fact, NGF, BDNF, and NT3 play central roles in the development of the nervous system via Trk and p75 neurotrophin receptors [38], which are associated with signaling pathways including PKC, CaMKII, AKT, and Erk. The signaling pathways mediate the proliferation and differentiation of neuronal precursor cells and also regulate neurotransmitter release, long-term potentiation, axonal and dendritic growth and guidance, and synaptic plasticity. Such decreased productions of GFs/NFs and their signaling molecules in AD animals were fully recovered after the transplantation of F3 and F3.ChAT NSCs, indicating the neuroprotective activities of NSCs (Figure 5A,B; Supplementary Figure S4).

It is of interest to note that the increased expression of CCL11, a chemokine that suppresses neurogenesis, in AD mice [39] was markedly reduced by F3.ChAT cell transplantation (Figure 5C). The CCL11-inhibiting activity of NSCs might have increased the number of nestin-positive NSCs exhibiting Ki-67 immunoreactivity in the hippocampus, confirming that the host NSCs are induced to proliferate (Figure 5D). In our previous study, such neuroregenerative phenomena were also observed in aging rats following the transplantation of amniotic membrane- or adipose tissue-derived stem cells [40], which was anticipated from reports that stem cell transplantation increased neurogenesis and neuronal differentiation in the hippocampus and subventricular zone of normal and AD animals [41,42]. Therefore, it is suggested that the restoration of MAP2 and cholinergic system of AD mouse brain by the transplantation of F3.ChAT human NSCs resulted in the increased neuroregeneration, in addition to the neuroprotective action of GFs/NFs [10,43,44].

To date, the administration of AChE inhibitors has been used for AD therapy to increase ACh concentration in the brain [1,3]. Since these drugs are only palliative, other therapeutic approaches including stem cell-based therapy are expected to replace the drugs. Several studies demonstrated the effectiveness of stem cells secreting neuroprotective GFs/NFs or modulating immune responses in AD animals [13,25,37]. However, it is believed that the ideal therapeutic strategy for AD should include the recovery of cognitive function and physical activity, neuroprotection against progressive tissue destruction, and neuroregeneration [10]. Recent studies of ours have demonstrated that the F3.ChAT human NSCs substantially restored the learning and memory functions in AD model and aging animals by increasing brain ACh levels [6,7,9]. In the present study, F3.ChAT cells exerted additional effects in APPswe/PS1dE9 double mutant animals, i.e., improved physical activity, restored MAP2 and cholinergic system, decreased Aβ deposition, inhibited inflammatory response, and induced proliferation in host stem cells. Such multiple faceted activities of F3.ChAT NSCs are induced by the secretion of ACh and GFs/NFs, the normalization of microglial function, and the inhibition of neurogenesis-suppressing chemokines by the stem cells. This study provides proof-of principle that the NSC-based gene therapy providing ChAT protein could effectively restore cognitive function in patients suffering with AD.

## 4. Materials and Methods

### 4.1. Ethics Statement

All procedures for experimental animals were approved by the Institutional Animal Care and Use Committee of Laboratory Animal Research Center (LARC) at Chungbuk National University (CBNU), Korea (approval No. CBNUA 221-1004). The animal experiments were carried out in accordance with the Standard Operation Procedures (SOP) of the institute (LARC at CBNU).

### 4.2. Human NSC Lines

The immortalized human HB1.F3 (F3) NSC line was prepared as described previously [13,45]. For the establishment of F3 NSC line overexpressing ChAT gene (F3.ChAT), F3 cells were infected with a retroviral vector encoding the human ChAT gene and selected for puromycin resistance [6,7]. ChAT mRNA expression and ChAT protein production were confirmed by reverse transcriptase-polymerase chain reaction (RT-PCR) using primers described in Supplementary Table S1 (Bioneer, Daejeon, Korea) and immunocytochemistry using ChAT-antibody (1:200, rabbit polyclonal, Chemicon, Temecula, CA, USA), respectively [6,7].

### 4.3. Animal Model and NSC Transplantation

APPswe/PS1dE9 transgenic (Tg) mice (*n* = 10/group) were obtained from Jackson Laboratory (Bar Harbor, ME, USA). The animals were maintained in a room with a constant temperature of 22 ± 2 °C, relative humidity of 55 ± 10%, and a 12-hour light/dark cycle, and fed standard rodent chow and purified water ad libitum. The genotyping for amyloid precursor proteins (APP) and presenilin 1 (PS1) was performed by PCR recommended by the Jackson Laboratory, using tissue samples from tail of mice. Eighteen-month-old wild-type (WT: Group 1) and Tg mice (Groups 2–4) were anesthetized with enfluorane and positioned in a sterotaxic frame. After incision of the skin and drilling a hole on the right skull under aseptic procedures, saline (5 μL: Group 2), F3 (Group 3) or F3.ChAT (Group 4) cells (10^6^ cells/mouse) were transplanted into the lateral ventricle at the following coordinates: posterior 1.0 mm, left lateral 2.0 mm, and ventral 3.0 mm from bregma [6,7,8,9]. The mice were subjected to learning and memory function tests 4 weeks after transplantation of the cells, and they were sacrificed for the analyses of distribution of transplanted cells in the brain, differentiation, and ChAT expression.

### 4.4. Cognitive Functions

Passive avoidance performance was assessed by Shuttle box (Med Associates, St. Albans, VT, USA) to evaluate memory acquisition and retention [6,7,9]. The Shuttle box apparatus consists of light and dark compartments; a light chamber equipped with a lamp and a dark chamber with a steel-grid floor for electric shock. On the trials, electric shock was delivered when mice entered the dark compartment from the light room through a guillotine door. The latency time of remaining in a room with the light on was recorded following electric shock (1 mA for 2 s) in a dark compartment. Nine consecutive trials at 5-min intervals were performed with electric shock when mice entered the dark compartment. The endpoint was set to 300 s, denoting the full acquisition of memory. A Morris water-maze performance was assessed in a round water bath (180 cm in diameter; Panlab Technology, Barcelona, Spain) filled with water (27 cm in depth) maintained at 22 ± 22 °C to evaluate spatial memory [6,7,9]. The bath was divided into four quadrants, and a hidden escape platform (10 cm in diameter, 25 cm in height) was submerged in the center of one quadrant, 2 cm below the surface of water. The mice were subjected to 3 trials a day for 9 consecutive days to find the hidden platform, based on several cues external to the maze. The endpoint was set to 300 s, if the animals failed to find the platform. Mean escape latency time, which were spent to escape onto the platform during trials, was recorded.

### 4.5. Neurobehavioral Functions

Spontaneous activities were evaluated using a video tracking system (Panlab Technology), connected to a CCTV monitor (Samsung, Changwon, Korea). Mice were placed in a quiet chamber and each time of the movement types—i.e., resting, slow-moving, and fast-moving—was recorded for 5 min following the 15-s adaptation time, and the ratio was analyzed [8,9,10,11]. Motor balance and coordination were evaluated using a rota-rod test system (Panlab Technology). Mice were placed on a rotating rod at a constant speed of 12 rpm, and the time for the mice to fall off the rod was recorded. The average latency was calculated from 3 consecutive measurements [6,7,8].

### 4.6. Analysis of Aβ Peptides in Brain Tissues

The levels of Aβ_1-40_ and Aβ_1-42_ peptides were measured from the hippocampal and cortical tissues. In brief, the brain (*n* = 5/group, left hemispheres) was removed after intracardial perfusion with cold saline, weighed, and homogenized in 5 volumes of Tris-buffered saline (TBS) containing a cocktail of protease inhibitors (Sigma-Aldrich, St. Louis, MO, USA). The samples were suspended in 2% sodium dodecyl sulfate (SDS) containing protease inhibitors and centrifuged at 100,000× *g* for 60 min, and the supernatant fraction was collected for enzyme-linked immunosorbent assay (ELISA) of soluble Aβ peptides. The remaining SDS-insoluble pellet was dissolved in 70% formic acid and centrifuged at 100,000× *g* for 60 min, and the supernatant fraction was collected for the assay of insoluble Aβ peptides. The SDS-soluble and SDS-insoluble levels of Aβ_1-40_ and Aβ_1-42_ peptides were analyzed using ELISA kits (Invitrogen, Carlsbad, CA, USA) according to the manufacturer’s instructions [4].

### 4.7. Analysis of ACh in Brain Tissues

The brain tissues (*n* = 5/group, right hemispheres) were weighed and homogenized in 9 volumes of phosphate-buffered saline containing a cocktail of protease inhibitors (Sigma-Aldrich). After centrifugation at 13,500 rpm for 6 min at 4 °C, the supernatant was measured for ACh concentration with the Amplex Red acetylcholine/acetylcholinesterase assay kit (Molecular Probes, Eugene, OR, USA) according to the manufacturer’s instructions [6,7,8,9]. In this assay, ACh is hydrolyzed by AChE to release choline, which is then oxidized by choline oxidase to betaine and H_2_O_2_. H_2_O_2_ interacts with Amplex Red (7-dihydroxyphenoxazine) in the presence of horseradish peroxidase to generate the highly fluorescent resorufin. The resulting fluorescence was measured in a fluorescence microplate reader using excitation in the range of 530–560 nm and emission at 590 nm.

### 4.8. RT-PCR Analysis in NSCs and Brain Tissues

Total RNA was extracted from NSC cultures and brain using TRIzol (Invitrogen). Complimentary DNA templates were prepared from 1 µg of total RNA primed with oligodT primers using 40 U of Moloney Murine Leukemia Virus reverse transcriptase (Promega, Madison, WI, USA) followed by 40 PCR cycles, and RT-PCR products were separated electrophoretically on an 1.2% agarose gel containing ethidium bromide [6,7,9]. The primers used for RT-PCR were described in Supplementary Table S1.

### 4.9. Western Blot Analysis in Brain Tissues

Mouse brain (*n* = 5/group, left hemispheres) was homogenized in RIPA cell lysis buffer (Sigma-Aldrich) with protease inhibitors. Proteins were obtained by centrifugation at 15,000 rpm at 4 °C for 15 min and quantified by the BCA protein assay kit (Pierce, Rockford, IL, USA). Proteins were denatured by heating for 5 min at 95 °C in 0.5 M Tris-HCl buffer (pH 6.8) containing 10% SDS and 10% ammonium persulfate, separated by electrophoresis on SDS-polyacrylamide gel, and transferred onto a polyvinylidene difluoride membrane in 25 mM Tris buffer containing 15% methanol, 1% SDS, and 192 mM glycine. After blocking for 2 h with 5% skim milk in TBS-Tween (TBS-T; 20 mM Tris, 137 mM NaCl, 0.1% Tween 20, pH 7.6), the membrane was incubated with antibodies specific for brain-derived neurotrphic factor (BDNF; 1:500, rabbit polyclonal, Santa Cruz Biotechnology, Santa Cruz, CA, USA), nerve growth factor (NGF; 1:500, rabbit polyclonal, Santa Cruz Biotechnology), ciliary neurotrophic factor (CNTF; 1:500, rabbit polyclonal, Santa Cruz Biotechnology), neurotrophin 3 (NT3; 1:500, goat polyclonal, Santa Cruz Biotechnology), glial cell-derived neurotrophic factor (GDNF; 1:500, rabbit polyclonal, Santa Cruz Biotechnology), tropomyosin-related kinase B (TrkB; 1:500, rabbit polyclonal, Santa Cruz Biotechnology), p-AKT1/2/3 (1:500, mouse monoclonal, Santa Cruz Biotechnology), calcium/calmodulin-dependent protein kinase II (CaMKII; 1:500, mouse monoclonal, Santa Cruz Biotechnology), protein kinase C (PKC; 1:500, mouse monoclonal, Santa Cruz Biotechnology), extracellular signal-regulated kinase (p-Erk; 1:500, rabbit polyclonal, Santa Cruz Biotechnology), β-site amyloid precursor protein-cleaving enzyme (BACE; 1:500, rabbit polyclonal, Santa Cruz Biotechnology) or neprilysin (1:500, rabbit polyclonal, Santa Cruz Biotechnology) overnight at 4 °C. After washing with TBS-T, the membrane was incubated with a secondary goat anti-rabbit IgG (1:2000, Santa Cruz Biotechnology), rabbit anti-goat IgG (1:2000, Santa Cruz Biotechnology) or goat anti-mouse immunoglobulin G (IgG; 1:2000, Santa Cruz Biotechnology) conjugated with horseradish peroxidase for 2 h at room temperature [9,10]. Then, the membrane was developed using an ECL solution (Pierce).

### 4.10. Immunohistochemistry in Brain Sections

The mouse brain (*n* = 5/group, right hemispheres) was perfusion-fixed with 10% paraformaldehyde solution and post-fixed in the same solution for 48 h, followed by cryoprotection in 30% sucrose for 72 h. Coronal cryosections in 30-μm thickness 1.0 mm posterior to bregma were prepared and processed for double immunostaining of human mitochondria (hMito; for human cells) and ChAT, neurofilament-high molecular weight protein (NF-H; for neurons) or glial fibrillary acidic protein (GFAP; for astrocytes) using antibodies specific for hMito (1:200, mouse monoclonal, Chemicon), ChAT (1:200, rabbit polyclonal, Chemicon), NF-H (1:200, rabbit polyclonal, Chemicon) or GFAP (1:200, rabbit polyclonal, Chemicon). Separately, amyloid plaques, activated microglia, host stem cells, and cell proliferation were confirmed by staining with antibodies for Aβ (1:200, mouse monoclonal, Chemicon), CD11b (1:200, rabbit polyclonal, Abcam, Cambridge, MA, USA), nestin (1:200, mouse monoclonal, Chemicon), and Ki-67 (1:200, rabbit polyclonal, Chemicon), respectively. Brain sections were incubated with the primary antibodies overnight at 4 °C, followed by secondary antibodies conjugated with Alexa Fluor-488 or -594 (1:400, Molecular Probes) for 2 h at room temperature [6,7,8,9], and then stained with 4′,6-diamidino-2-phenylindole (DAPI) for 30 min. All the samples were evaluated immediately after staining and photographed with a laser-scanning confocal microscope (Zeiss, New York, NY, USA).

From the entire microscopic field of brain sections (*n* = 5/group, right hemispheres), the hMito-, NF-H-, GFAP-, and ChAT-positive cells were counted. The number and ratio of human cells survived, differentiated into neurons or astrocytes, and producing functional protein (ChAT) were calculated from the immunostained cells.

### 4.11. Statistical Analysis

Data are presented as mean ± standard error of the mean. The statistical significance between group comparisons was determined by one-way analysis of variance (ANOVA), followed by post-hoc Tukey’s multiple comparison test using the SAS program (version 6.12; SAS Institute, Inc., Cary, NC, USA, http://www.sas.com). Separately, passive avoidance and water-maze performances were analyzed by two-way ANOVA. *p*-values < 0.05 were considered to be statistically significant.

## 5. Conclusions

The present study demonstrated that neural stem cells overexpressing choline acetyltransferase gene improved complex cognitive and physical deficits of Alzheimer disease model mice by releasing acetylcholine, reducing Aβ deposit, and promoting neuroregeneration. It is suggested that F3.ChAT cells could be a candidate for the improvement of neurobehavioral and cognitive dysfunctions in CNS disorders such as AD, as a therapeutic strategy compared with the transient, preventive mode of chemical drugs.

## Supplementary Materials

Supplementary materials can be found at https://www.mdpi.com/1422-0067/21/11/3958/s1.
