# Supplementary material for: Human Neural Stem Cells Encoding ChAT Gene Restore Cognitive Function via Acetylcholine Synthesis, Aβ Elimination, and Neuroregeneration in APPswe/PS1dE9 Mice
Source: Int J Mol Sci. 2020 May 31;21(11):3958. doi: 10.3390/ijms21113958 (PMC7313059; doi:10.3390/ijms21113958)
Supplement: Supplementary file 1 [file ijms-21-03958-s001.pdf]

International Journal of
Molecular Sciences

MDPI

Supplementary Table 1. Primer sequences used for RT-PCR analysis

A. Primer sequences used for human NSCs			Accession No.
Gene	Primer	Sequences	
ChAT	Forward<br>Reverse	5'-CTGTGCCCCCTTCTAGAGC-3'<br>5'-CAAGGTTGGTGTCCCTGG-3'	NM_001142933
ChT1	Forward<br>Reverse	5'-ATCCCAGCCATACTCATTGG-3'<br>5'-CAGAAACTGCACCAAGACCA-3'	NM_021815
VAChT	Forward<br>Reverse	5'-ACGTGGATGAAGCATACG-3'<br>5'-CTGAGACATGGCGCACGT-3'	NM_003055
m1AChR	Forward<br>Reverse	5'-AGACGCCAGGCAAAGGGGGTGG -3'<br>5'-CACGGGGCTTCTGGCCCTTGCC-3'	NM_000738
nAChR α5	Forward<br>Reverse	5'-TCATGTAGACAGGTACTTC-3'<br>5'-ATTTGCCCATTTATAAATAA-3'	NM_000745
nAChR β2	Forward<br>Reverse	5'-CAGCTCATCAGTGTGCA-3'<br>5'-GTGCGGTCGTAGGTCCA-3'	NM_000748
AChE	Forward<br>Reverse	5'-GCTCGGCCGCCTCAGACGCCG-3'<br>5'-TCCTCGCTCAGCTCACGGTTGGG-3'	NM_000665
B. Primer sequences used for mouse brain tissues			
ChAT	Forward<br>Reverse	5'-GAGCGAATCGTTGGTATGACAA-3'<br>5'-AGGACGATGCCATCAAAAGG-3'	NM_009891
ChT1	Forward<br>Reverse	5'-GCAGCTTTTGGGTGCCTG-3'<br>5'-TGTGGAAGCTCCAATAGCTCC-3'	NM_022025
VAChT	Forward<br>Reverse	5'-GGGTCGGCTCGGTCAATC-3'<br>5'-CAAATAGCACGCCTATCTTCACAT-3'	NM_021712
m1AChR	Forward<br>Reverse	5'-CCCCTGGGTCACCTTCCT-3'<br>5'-TTCCCCGGGTTTCACTCTCT-3'	NM_007698
nAChR α5	Forward<br>Reverse	5'-CAACATCCACCACCGCTCTT-3'<br>5'-TTTGGGAAGCTTGTGGAGAAAT-3'	NM_176844
nAChR β2	Forward<br>Reverse	5'-TCGTCGCAAACCGCTCTT-3'<br>5'-GATGGCCAGCGAGGTGAT-3'	NM_009602
AChE	Forward<br>Reverse	5'-CCTGGATCCCTCGCTGAA-3'<br>5'-CCTGTGCGGGCAAAATTG-3'	NM_009599
TNF-α	Forward<br>Reverse	5'- GCACAGAAAGCATGATCCG-3'<br>5'- GCCCCCCATCTTTTGGG-3'	NM_013693
IL-1β	Forward<br>Reverse	5'- ACCTGTCCTGTGTAATGAAAGACG-3'<br>5'- TGGGTATTGCTTGGGATCCA-3'	NM_008361
CD11b	Forward<br>Reverse	5'- GTGTGACTACAGCACAAGCCG-3'<br>5'- CCCAAGGACATATTCACAGCCT-3'	NM_008401
SRA	Forward	5'- TGAACAAGAGGATGCTGACTG-3'	NM_031195

	Reverse	5'- GGAGGGGCCATTTTTAGTGC-3'	
SRB	Forward	5'- TTTGGAGTGGTAGTAAAAAGGGC-3'	NM_016741
	Reverse	5'- TGACATCAGGGACTCAGAGTAG-3'	
RAGE	Forward	5'- ACTACCGAGTCCGAGTCTACC-3'	NM_007425
	Reverse	5'- GTAGCTTCCCTCAGACACACA-3'	
CD36	Forward	5'- GAACCACTGCTTTCAAAAACTGG-3'	NM_007643
	Reverse	5'- TGCTGTTCTTTGCCACGTCA-3'	

ChAT, choline acetyltransferase; ChT1, choline transporter; VAChT, vesicular acetylcholine transporter; m1AChR, muscarinic 1 acetylcholine receptor; nAChR, nicotinic acetylcholine receptor; AChE, acetylcholinesterase; TNF-α, tumor-necrosis factor-α; IL-1β, interleukin-1β; SR, scavenger receptor; RAGE, receptor for advanced glycation end products.

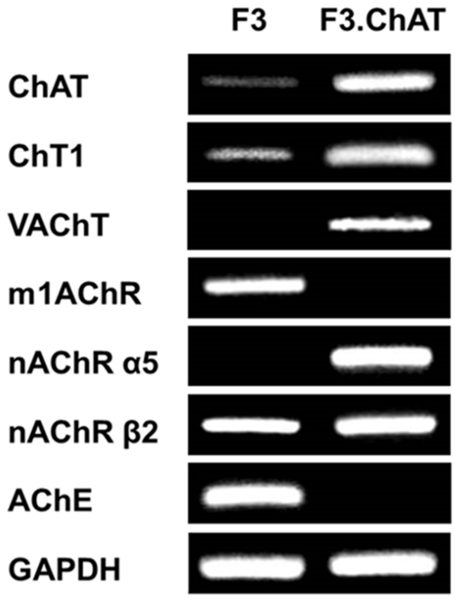

Supplementary Figure 1. Cholinergic nervous system markers in human neural stem cells (NSCs). Cholinergic markers in F3 and F3.ChAT NSCs were demonstrated by RT-PCR. ChAT: choline acetyltransferase, ChT1: choline transporter, VAChT: vesicular acetylcholine transporter, m1AChR: muscarinic 1 acetylcholine receptor, nAChR: nicotinic acetylcholine receptor, AChE: acetylcholinesterase, GAPDH: glyceraldehyde-3-phosphate dehydrogenase.

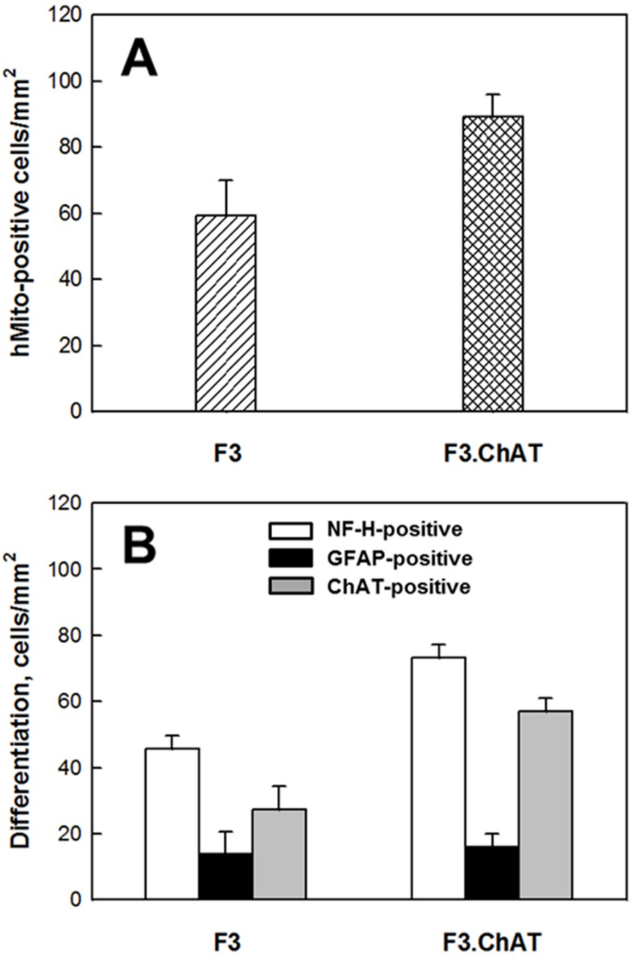

Supplementary Figure 2. Number of NSCs survived (A) and differentiated into neurons and astrocytes, and expressing ChAT protein (B) in the mouse brain environment of APPswe/PS1dE9 mice 4 weeks post-transplantation (1 x 10^6 cells/mouse).

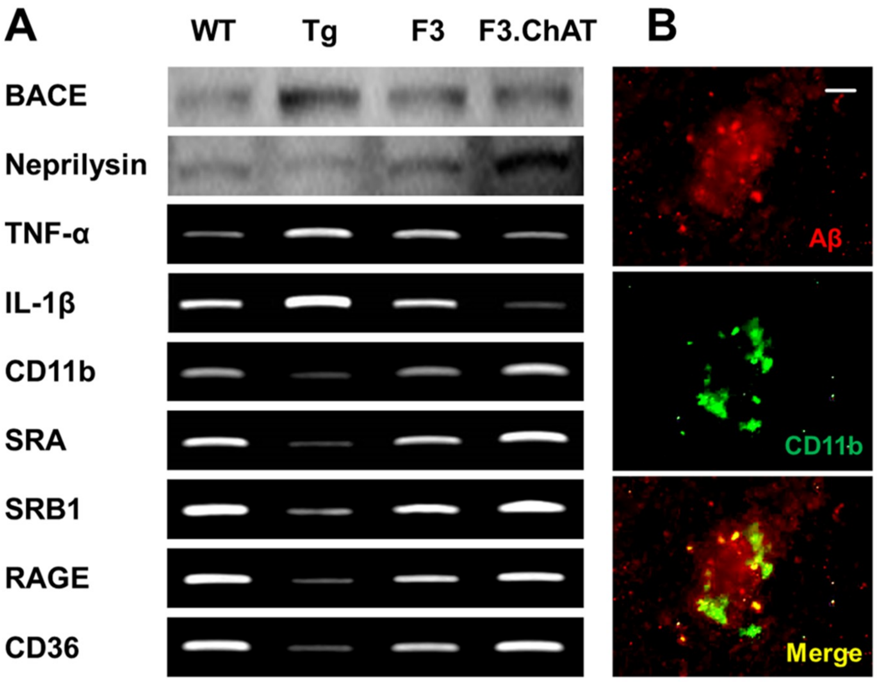

Supplementary Figure 3. Microglial function in the brain of wild-type (WT) and APPswe/PS1dE9 transgenic (Tg) mice transplanted with F3 or F3.ChAT neural stem cells (NSCs). (A): Microglial functions related to Aβ formation and degradation, cytokine secretion, and Aβ scavenging. (B): A representative finding of CD11b-positive microglia surrounding amyloid plaques in the hippocampus of F3.ChAT NSC-transplanted APPswe/PS1dE9 Tg mouse. Bar = 10 μm

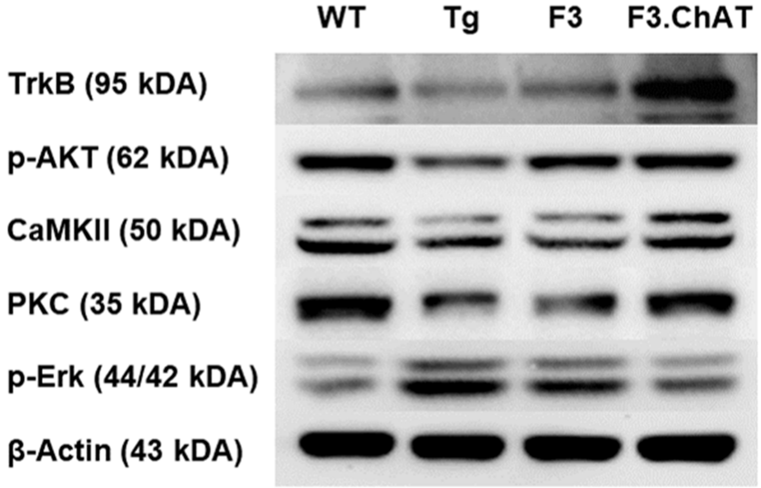

Supplementary Figure 4. Production of representative signaling molecules of growth and neurotrophic factors in the brain of wild-type (WT) and APPswe/PS1dE9 transgenic (Tg) mice transplanted with F3 or F3.ChAT neural stem cells.
